# Patellofemoral Joint (PFJ) Arthroplasty in Young Patients: Mid-term Results and Functional Outcomes

**DOI:** 10.7759/cureus.93062

**Published:** 2025-09-23

**Authors:** Mohamed Elmubark, Kars P Valkering, Wim E Tuinebreijer, Aysha Rajeev

**Affiliations:** 1 Trauma and Orthopaedics, Gateshead Health NHS Foundation Trust, Gateshead, GBR; 2 Trauma and Orthopaedics, Bergman Clinics, Rijswijk, NLD; 3 Surgery, Erasmus University Medical Center, Rotterdam, NLD

**Keywords:** arthritis, arthroplasty, outcomes, patellofemoral, young adults

## Abstract

Purpose

Isolated patellofemoral arthritis is a well-recognized variant of knee osteoarthritis. The cause of isolated patellofemoral arthritis can be traumatic, secondary to malalignment, degenerative, or idiopathic. This study aims to evaluate whether the results of our third-generation patellofemoral arthroplasty (PFA) support its continued use as a viable treatment option for isolated patellofemoral arthritis.

Methods

A retrospective cohort study was conducted using the electronic NHS trust database to identify all patients who underwent PFA between September 2008 and April 2014. Each patient was subsequently reviewed at our outpatient clinic, where they completed a series of standardized questionnaires to assess treatment outcomes. These included the Short Form 12v2, the Forgotten Joint Score (FJS), the Kujala Score, and the Knee Injury and Osteoarthritis Outcome Score (KOOS). A total of 44 patellofemoral joints (PFJs) were included in the study.

Results

Four patients required revision surgery to convert their PFJ replacement to a total knee replacement. Additionally, three patients underwent arthroscopy for the management of patella maltracking. When asked about their satisfaction and whether they would choose to undergo the same surgery again, only seven patients indicated that they would opt for the procedure again. The FJS revealed that the majority of patients (85%) reported being rarely or never aware of their artificial joint in daily activities. The average Kujala score was found to be 4, reflecting moderate discomfort or dysfunction levels. The KOOS indicated that most patients experienced mild to moderate symptoms, with occasional bouts of pain and moderate stiffness. Importantly, the KOOS findings also suggested that the patients’ daily activities were not severely impacted.

Conclusion

PFJ arthroplasty can yield positive outcomes for younger patients who suffer from isolated patellofemoral arthritis. However, achieving successful results is heavily influenced by the careful and appropriate selection of patients rather than the choice of the implant itself.

## Introduction

Isolated patellofemoral arthritis, characterized by the selective involvement of the patellofemoral joint (PFJ) with relative sparing of the tibiofemoral joint, is a well-recognized variant of knee osteoarthritis, affecting approximately 10% of patients with knee arthritis [1-3]. This condition tends to be more prevalent in younger patients and occurs more frequently in females than in males [3]. The aetiology of isolated patellofemoral arthritis can be traumatic, secondary to malalignment, degenerative, idiopathic, or a combination of these factors [4].

When conservative treatment fails (exercise, physical therapy, taping, and injections), treatment options become limited. Surgical interventions include patellectomy [4,5], debridement [5], extensor mechanism realignment (Maquet procedure) [5], isolated patella resurfacing [4], and lateral facetectomy [5], though these procedures often yield unpredictable and generally suboptimal outcomes. In contrast, total knee arthroplasty (TKA) has shown predictable, favourable results for isolated patellofemoral osteoarthritis, with or without patellar resurfacing [6-9]. However, isolated patellofemoral arthroplasty (PFA) has gained increasing attention as it preserves the femorotibial joint and maintains joint kinematics [10].

This study aims to evaluate whether the results of our third-generation PFA (prosthetic designs with gender specific features, enhanced geometry, and reduced overhang) support its continued use as a viable treatment option for isolated patellofemoral arthritis.

## Materials and methods

A retrospective cohort study was conducted using the electronic NHS trust database to identify all patients who underwent PFA between September 2008 and April 2014. A consecutive series of patients treated with PFA during this period was included in the analysis. Each patient was subsequently reviewed at our outpatient clinic, where they completed a series of standardized questionnaires designed to assess treatment outcomes. These included the Short Form 12v2 (SF-12) [11], the Forgotten Joint Score (FJS) [12], the Kujala Score [13,14], and the Knee Injury and Osteoarthritis Outcome Score (KOOS) [15]. In addition to these validated outcome measures, patients were also asked to provide subjective feedback regarding their symptoms, specifically whether they experienced an improvement in knee pain or function following PFA, and whether they would choose to undergo the same procedure again. This was a service evaluation rather than a clinical trial, so full ethical approval was not required. However, the study was registered with the research and development department of the local NHS trust to ensure compliance with institutional guidelines and oversight.

**Figure 1:**
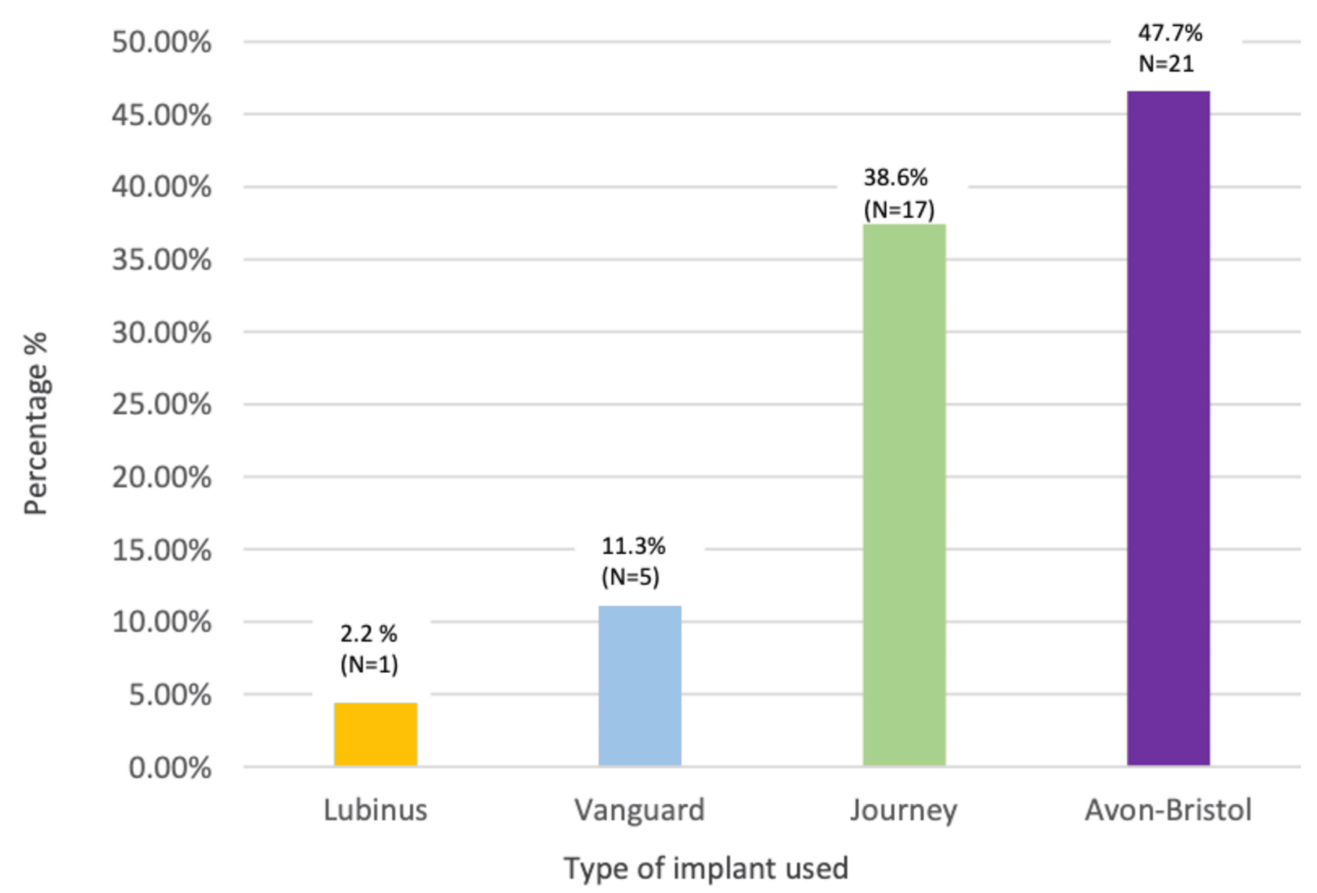
Types of implant used

**Figure 2:**
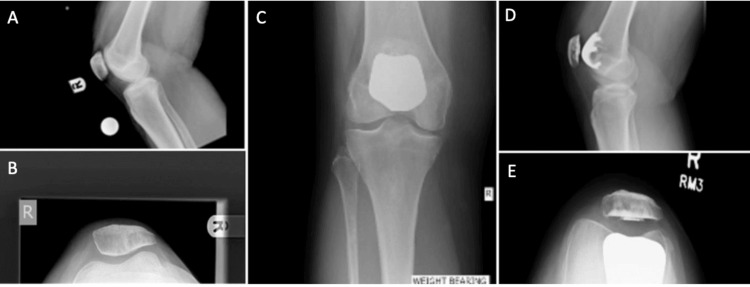
Pre-op and post-op PFJR with Avon prosthetic (A) Pre-operative lateral view knee X-ray (B) Pre-operative skyline view knee X-ray (C) Postoperative AP weight-bearing view knee X-ray showing Avon prosthesis (D) Postoperative lateral view knee X-ray showing Avon prosthesis (E) Postoperative skyline view knee X-ray showing Avon prosthesis PFJR: patellofemoral joint replacement; AP: anteroposterior

**Figure 3:**
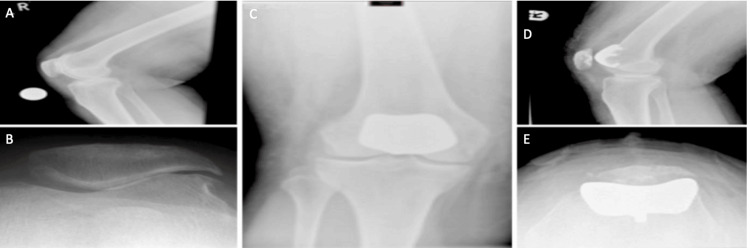
Pre-op and post-op PFJR with Journey prosthesis (A) Pre-operative lateral view knee X-ray (B) Pre-operative skyline view knee X-ray (C) Post-operative AP weight-bearing view knee X-ray with Journey prosthesis (D) Post-operative lateral view knee X-ray with Journey prosthesis (E) Post-operative skyline view knee X-ray with Journey prosthesis PFJR: patellofemoral joint replacement; AP: anteroposterior

Four distinct types of prosthetic devices were employed in this cohort (Figure 1): Avon-Bristol (Stryker Howmedica Osteonics, Allendale, NJ, USA) (Figures 2C-2E), Journey (Smith+Nephew, UK) (Figures 3C-3E), Vanguard Patellofemoral Replacements (PFR) (Zimmer Biomet, USA), and Lubinus (Waldemar Link GmbH & Co. KG, Hamburg, Germany). Twenty-one patients received the Avon-Bristol implant, 17 were implanted with the Journey prosthesis, five were fitted with the Vanguard PFR, and one patient was treated with the Lubinus implant.

All patients included in the study were followed closely throughout their postoperative recovery, with formal assessments conducted two years following the surgical intervention. These evaluations were performed to measure the effectiveness and outcomes of the surgery using several established and validated scoring systems. The specific instruments used included the SF-12 for general health-related quality of life (QOL), the FJS to assess joint awareness in daily life, the Kujala Patellofemoral Score to evaluate patellofemoral-specific symptoms and function, and the KOOS to measure knee-specific health and QOL in patients with osteoarthritis or similar knee conditions. These comprehensive outcome measures provided a detailed picture of the patient's recovery and long-term functional results following PFA.

Statistical analysis

The data were analyzed using standard descriptive statistical methods, with all variables summarized through means and standard deviations to provide an overview of the patient cohort's characteristics. To assess potential factors that could influence the outcomes of PFA, a range of variables was considered. These included patient demographics (such as age and gender), clinical factors (such as body mass index (BMI)), and aspects related to the surgical procedure, including the type of implant used, the surgeon performing the procedure, the side of the knee operated on (unilateral or bilateral), as well as any comorbid conditions. Specifically, we evaluated the impact of factors such as profession, use of steroids, rheumatoid arthritis, diabetes mellitus, smoking habits, and alcohol consumption on the surgical outcomes. Statistical significance for all tests was determined using a threshold of p < 0.05, and all p-values were two-tailed to account for the possibility of effects in both directions. To ensure robust and accurate analysis, all statistical procedures were carried out using IBM SPSS Statistics for Windows, Version 22 (Released 2015; IBM Corp., Armonk, New York, United States). This approach allowed for a comprehensive examination of the relationship between various clinical and demographic factors and the overall outcomes of PFA in this cohort.

## Results

The study included 44 joints, with 68% (30) being females and 32% (14) being males. The mean age was 57 years (SD = 12.7), and the mean BMI was 30.1 kg/m^2^ (SD = 6.0). The age of the patients varied, with the youngest being 32 years old and the oldest 53 years old, thus representing a relatively narrow age range of 32 to 53. The average postoperative time for completing questionnaires was 2.53 years (SD = 1.8).

Regarding the side of surgery, 57% (25) had procedures on the right side, while 43% (19) had left-side operations. Most participants (84%, 37) did not require reoperation, although 16% (7) did (Table 1). A total of 9% (4) of patients had their procedure converted to TKA. For prosthesis types, 38.6% (17) had the Journey prosthesis, 47.7% (21) had the Avon-Bristol prosthesis, 11.3% (5) had the Vanguard prosthesis, and 2.2% (1) had the Lubinus prosthesis. A total of 25% (11) of patients had steroid injections before surgery.

**Table 1:** Patient characteristics (N=44) N: sample size; SD: standard deviation; TKA: total knee arthroplasty

Characteristics	N (%)/Mean (SD)
Gender	
Female	30 (68)
Male	14 (32)
Age in years, mean (SD)	57 (12.7)
BMI (kg/m^2^), mean (SD)	30.1 (6.0)
Postoperative time in years of filling in questionnaires, mean (SD)	2.53 (1.8)
Side	
Right	25 (57)
Left	19 (43)
Reoperation	
No	37 (84)
Yes	7 (16)
Converted to TKA	4 (9)
Prosthesis	
Journey	17 (38.6)
Avon-Bristol	21 (47.7)
Vanguard	5 (11.4)
Lubinus	1 (2.3)
Steroid use	11 (25)

The functional score at the final follow-up showed a KOOS pain score of 55.1 (SD 26.8), symptoms score of 47.6 (SD 18.3), activities of daily living (ADL) score of 55 (SD 25.4), sports/recreation score of 29.3 (SD 29.3), and QOL score of 40.4 (SD 29.2). The SF-12 physical component score was 34.6 (SD 11.1) and the mental component was 44.2 (SD 11.3). The Kujala score was 55.1 (SD 21.5) and the FJS was 21.9 (SD 20.5) (Table 2).

**Table 2:** Results after mean follow-up SD: standard deviation; KOOS: Knee Injury and Osteoarthritis Outcome Score; SF-12: Short Form-12 Health Survey; ADL: activities of daily living

Scales	Mean (SD)
Forgotten Joint Score	21.9 (20.5)
Kujala Patellofemoral Score	55.1 (21.5)
KOOS Pain Score	55.1 (26.8)
KOOS Symptoms Score	47.6 (18.3)
KOOS ADL Score	55.0 (25.4)
KOOS Sports/Recreation	29.3 (29.3)
KOOS Quality of Life	40.4 (29.2)
SF-12 Physical Component Score	34.6 (11.1)
SF-12 Mental Component Score	44.2 (11.3)

Twenty-five percent of patients had steroid injections into the PFJ previously. Patients who used steroids in the past showed significantly poorer outcomes in several areas. Steroid users had significantly lower scores, indicating worse patellofemoral function (Kujala Patellofemoral Score, p = 0.028). Steroid users reported significantly lower participation in sports and recreation, suggesting that steroid use impacts functional mobility (KOOS Sports/Recreation Score, p < 0.001). Although not statistically significant, steroid users had a lower physical health score, indicating potential negative effects on overall physical health (SF-12) (Physical Component Score, p = 0.070). In contrast, no significant differences were observed in the KOOS Pain Score (p = 0.232), KOOS Symptoms Score (p = 0.130), or SF-12 Mental Component Score (p = 0.674), suggesting that steroid use does not significantly affect pain perception or mental health outcomes (Table 3).

**Table 3:** Results of questionnaires comparing steroid use versus no steroid use SD: standard deviation; P-value: probability value; KOOS: Knee Injury and Osteoarthritis Outcome Score; SF-12: Short Form-12 Health Survey; ADL: activities of daily living

Scales	No, Mean (SD)	Yes, Mean (SD)	P-value
Forgotten Joint Score	26.0 (20.0)	12.1 (18.9)	0.056
Kujala Patellofemoral Score	60.0 (21.6)	42.6 (15.9)	0.028
KOOS Pain Score	58.1 (27.2)	46.7 (25.0)	0.232
KOOS Symptoms Score	50.2 (19.5)	40.4 (12.8)	0.130
KOOS ADL Score	57.6 (26.5)	48.0 (21.3)	0.286
KOOS Sports/Recreation	36.3 (30.7)	10.2 (12.6)	<0.001
KOOS Quality of Life	55.0 (28.1)	29.5 (30.4)	0.145
SF-12 Physical Component Score	36.6 (11.3)	29.2 (8.8)	0.070
SF-12 Mental Component Score	43.7 (11.7)	45.5 (10.8)	0.674

**Figure 4:**
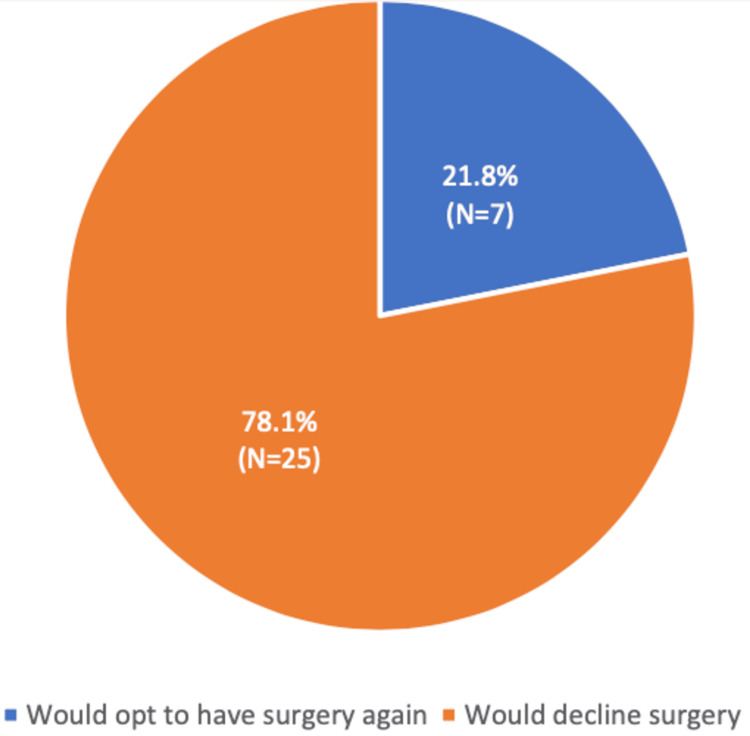
Patient satisfaction rates (%)

When asked about their satisfaction with the procedure and whether they would choose to undergo the same surgery again, only seven patients (21.8%) indicated that they would opt for the procedure a second time (Figure 4).

**Figure 5:**
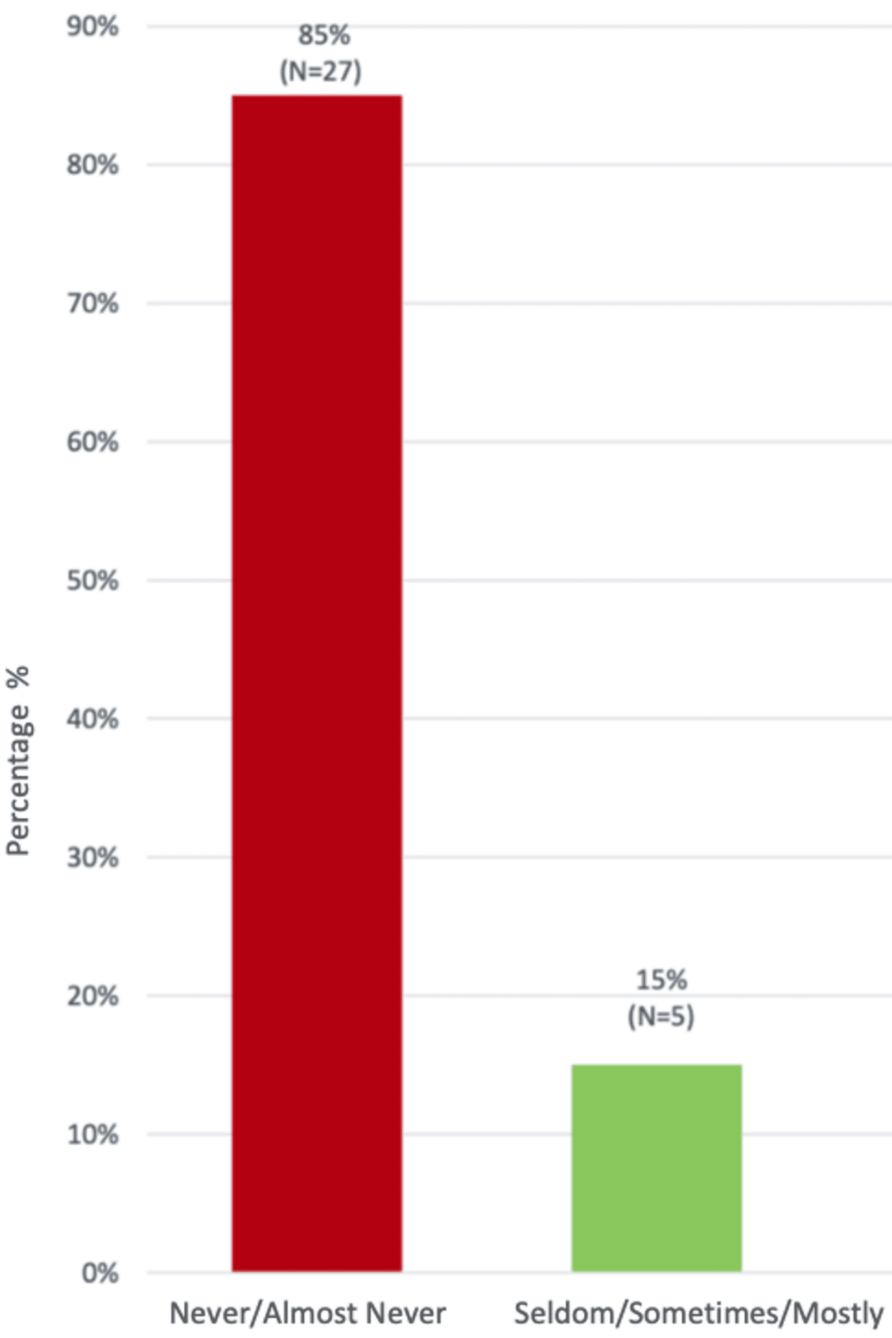
Forgotten Joint Score (FJS) showing level of joint awareness in percentage (%)

The FJS revealed that the majority of patients, approximately 85%, reported being rarely or never aware of their artificial joints in daily activities, indicating a generally favourable outcome in terms of joint awareness and function (Figure 5). The overall impact on their QOL and daily functioning was relatively modest.

## Discussion

The study underscores the significant impact of factors such as steroid use, BMI, and surgical interventions (bilateral vs. unilateral) on patient outcomes. Steroid use was associated with worse functional scores, particularly in terms of patellofemoral function and sports participation. While patients with bilateral operations reported more difficulties, this was not statistically significant. These findings highlight the importance of personalized care and monitoring of patients who have received steroid doses pre-operatively to optimize functional recovery and QOL for patients undergoing joint replacement procedures.

The study population was predominantly female (68%) with a mean age of 57 years, consistent with the typical age for joint replacement procedures. The mean BMI of 30.1 kg/m^2^ indicates that many participants were overweight or obese, a known risk factor for PFJ arthritis. Most patients (84%) did not require reoperation, suggesting the initial success of the procedures. However, 9% of the cohort had a revision of PFJ to a total knee replacement (TKR) due to the progression of arthritis in the medial and lateral compartments. Patients who underwent bilateral operations had a higher FJS (33.6 vs. 17.8) (Table 4), suggesting more difficulty in "forgetting" joint problems. However, this difference was not statistically significant (p = 0.096), indicating that factors beyond the presence of bilateral operations, such as disease severity or postoperative care, may contribute to joint discomfort.

**Table 4:** Result of questionnaire for patients with bilateral operations SD: standard deviation

Scale	Bilateral No, Mean (SD)	Bilateral Yes, Mean (SD)	P-value
Forgotten Joint Score	17.8 (16.8)	33.6 (25.8)	0.096

The average Kujala score, which specifically measures patellofemoral symptoms and function, was found to be 4, reflecting moderate levels of discomfort or dysfunction in the cohort. The KOOS provided further insight into the overall health and QOL of the patients. The results indicated that most patients experienced mild to moderate symptoms, with occasional bouts of pain and moderate stiffness. Importantly, the KOOS findings also suggested that the patient’s daily activities were generally not severely impacted, with only mild to moderate limitations observed in terms of their ability to perform routine tasks. This suggests that while the patients reported some level of ongoing discomfort and functional impairment.

Since the introduction of PFA [16-19], early designs suffered from high complication rates due to design limitations and suboptimal surgical techniques [20,21]. Subsequent improvements in instrumentation and biomechanical understanding led to better outcomes with second-generation designs [20,22,23], and further advancements have been made with the current third-generation PFA implants [19,24,25]. Despite the growing popularity of PFA, high failure rates persist, with midterm success rates varying from 42% to 88% [26,11,12].

The ideal candidate for PFJ replacement is a patient who exhibits severe and debilitating symptoms and signs of isolated PFJ disease. Ackroyd et al. conducted a study on 109 consecutive patients who underwent PFJ arthroplasty with the use of Avon implants [2]. Their findings revealed a 4.2% failure rate within the first five years post-surgery, while the success rate, as measured by the Bristol Knee Scores, was 80% after five years of follow-up. Additionally, disease progression was observed in 28% of patients, and re-operation was required in eight knees, including nine instances where TKRs were performed at the 10-year follow-up.

Mohammed et al. reported on a series of 101 PFJ arthroplasties performed using the Lubinus, Avon, and FPV prosthetic systems, with a mean follow-up period of four years [27]. In their cohort, 35 patients required re-operation, and four patients underwent revision to TKR. Notably, all of the revisions occurred in patients who had received the Lubinus implant. Data from the Australian Joint Registry highlighted that the five-year revision rate for the Avon PFJ implants stood between 7.6% and 30.3% [16], suggesting a relatively higher rate of complications for this particular implant system.

In contrast, Odumenya et al. reported on a series of 50 Avon PFJ replacements, observing a remarkable 100% survival rate at five years [28], which contrasts with the findings of earlier studies and points to the potential benefits of advancements in implant design or surgical techniques. Our study shows a higher re-operation rate (12.5% of patients requiring revision to TKR). This is notably higher than Ackroyd et al.'s findings, where the failure rate was only 4.2%. However, the higher revision rate in our cohort could be attributed to variations in patient demographics or implant type. Our revision rate of 12.5% aligns more closely with the findings in the Australian Joint Registry for Avon implants [29]. The 100% survival rate at five years observed by Odumenya et al. is a significantly more favourable outcome compared to the 12.5% revision rate in our cohort. The difference in outcomes may be attributed to factors such as variations in surgical technique, patient selection criteria, or implant design refinements.

Although only 21% of patients would opt for the surgery again, it’s important to note that our cohort’s patients reported moderate to mild symptoms on the Kujala and KOOS scales, indicating some success in terms of symptom control, but not necessarily high patient satisfaction. Mohammed et al. did not specifically report on patient satisfaction in their study, but their findings regarding re-operations suggest a relatively high rate of issues with some implants [27]. The fact that only 21% of our patients would choose the same procedure again, despite relatively good objective scores (such as the FJS and KOOS), suggests that patient satisfaction is a significant concern, likely influenced by ongoing symptoms or complications that may not be fully captured by clinical scores. Our findings on functional outcomes, particularly the FJS, indicate that while the joint itself might be functionally adequate for most patients (as they report being unaware of it in daily activities), the level of discomfort as measured by the Kujala and KOOS scores shows that many patients still experience moderate symptoms. This suggests that while our cohort's functionality is reasonably preserved, there remains a gap between symptom relief and overall patient satisfaction.

Our revision rate (12.5%) is higher than that reported by Ackroyd et al. and Odumenya et al. (100% survival). This difference may suggest a need to re-evaluate patient selection criteria or surgical techniques. The relatively high rate of revision to TKR in our cohort may be indicative of ongoing joint dysfunction, and our results might imply a need for more stringent guidelines when considering PFJ replacement in patients.

Limitations of the study

**Figure 6:**
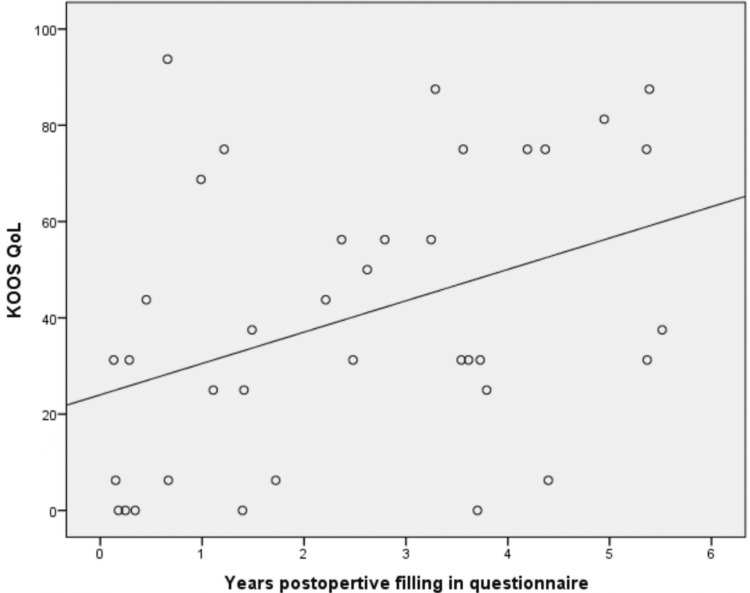
Postoperative KOOS QOL scores over time (years) KOOS QOL: Knee Injury and Osteoarthritis Outcome Score quality of life

The limitation of our study is that it is a retrospective analysis with a sample size of 44 patients. The study used four different implants, which may have contributed to the varied functional outcomes. The average follow-up period was also short, as we were able to collect post-operative scores for a period of up to six years (Figure 6).

## Conclusions

PFJ arthroplasty can yield positive and promising outcomes for younger patients who suffer from isolated patellofemoral arthritis, as it provides a targeted approach to addressing the specific area of joint degeneration. However, achieving successful results in such cases is heavily influenced by the careful and appropriate selection of patients rather than the choice of the implant itself.
